# The Medieval Attitude to Science

**Published:** 1918-02-16

**Authors:** 


### GLIMPSES BACKWARD.

### III. The Medieval Attitude to Science.

In considering, as we have done in the two preceding articles of this series, some of the more signal achievements of bygone masters of medicine, the reader may perhaps have been struck by the attitude of independent research, so greatly in contrast to what is popularly conceived as being the spirit of the Middle Ages.

But a moment's reflection will establish the essential similarity between the great teachers of fourteenth-century Italy and the scientific investigators of our own day. The mass of mankind, of all ages and of all creeds, is at bottom credulous. Modern science is as much the affair of a few persons and as much obsessed by fetishes as ever were the speculations of the medieval scholiasts. To-day we are obsessed by toxins, and every disease whose pathology is obscure is attributed to a condition of toxæmia, the modern equivalent of the evil humours and pestilent vapours at which we smile in the pages of old black-letter folios.

### Albertus Magnus and his Pupils.

Certainly one of the most fascinating of pursuits is to trace among the voluminous writings of these old-time Churchmen, scholars, and alchemists, the strange previsions and anticipations of modern thought that glimmer and flash with unexpected brilliance amid a wilderness of arid speculation. It is commonly supposed that in the Middle Ages little was done in the way of original research, and that the teachings of the ancient philosophers, Aristotle, Galen, Hippocrates, and the rest, were accepted without question. There is, of course, a basis of truth in such a supposition. But the thirteenth, and to a greater extent the succeeding centuries, produced many minds capable of brilliant observation and no less brilliant deduction. Thus at the University of Paris shortly after the middle of the thirteenth century were gathered three of the most remarkable minds that have ever figured together—Albertus Magnus, and his two illustrious pupils, Roger Bacon, the Franciscan, and St. Thomas of Aquin. To deal adequately with the achievements of any one of these men would take far longer than the space at our disposal permits, but it will nevertheless be of value to consider some of their deductions in the light of modern knowledge.

### Roger Bacon's Prophecies.

Roger Bacon, for instance, in a well-known passage of the "Opus Magnum," predicts that "One may cause to burst forth from bronze, thunderbolts more formidable than those produced by nature. A small quantity of prepared matter occasions a terrible explosion accompanied by a brilliant light. One may multiply this phenomenon so far as to destroy a city or an army." Realising, moreover, that such a power would in time to come be harnessed for the service of man, he goes on to say, "Art can construct instruments of navigation, such that the largest vessels, governed by a single man, will traverse rivers and seas more rapidly than if they were filled with oarsmen. One may also make carriages which without the aid of any animal will run with remarkable swiftness." His invention of the telescope is, in point of fact, apocryphal, but he did much to establish the theory of lenses upon a mathematical basis. Marvellous as it may seem, he taught that light travelled at a definite speed, though at such a rate that it would be impossible to measure it by any terrestrial means. As it was not for another three centuries that Römer, the German astronomer, demonstrated the actual speed of light by his observations on the planet Jupiter when eclipsing its moons, such a deduction is indeed extraordinary. He also predicted that in time to come men would be able to fly, though for this purpose he conceived a windlass worked by hand which might generate sufficient force to raise a man from the earth.

### The Philosopher's Stone.

St. Thomas Aquinas, whose "Summa Theologiæ" was an epitome of all the science of the period, was, not unnaturally, a distinguished physicist. He taught, for example, a belief in the indestructibility of matter. "*Nihil omnino in nihilum redigetur*" (Nothing at all will ever be reduced to nothingness) he said at the conclusion of a series of lectures on Cosmology at the University of Paris, and by some uncanny prevision he seems to have anticipated by well-nigh seven centuries the latest doctrines of the composition of matter. He declared that in Nature there were two essential principles, prime matter and form. By *forma* he meant the dynamic element in matter, whilst by *materia prima* he meant the underlying substratum of material, the same in every substance, but differentiated by the dynamics of matter. This philosophical theory gave the basis for the idea that one element could be changed into another if only some substance were discovered which would change its *forma* (we should say the rhythm of its molecules) into that of another element. Such a substance was the philosopher's stone, in the search for which the medieval chemists laid the foundations of modern chemical knowledge. A quarter of a century ago such an idea would have been ridiculous. But now that Sir William Ramsay has succeeded in changing radium into helium, and the elements are admitted not to be elements at all, but only different stages of evolution of the *materia prima*, we cannot afford to scoff at such theories. The elixir of life, it is true, still remains to be discovered, Metchnikoff and the lactic acid bacillus notwithstanding, but

☛ Previous articles appeared on January 5, page 285, and January 19, page 335.

**GLIMPSES BACKWARD**—(*continued*).

who shall say that the schoolmen were not as wise as we?

The Greatest of the Alchemists.

At rare and uncertain periods in the world's history there is born a man whose intellectual attainments so completely overshadow his own and succeeding generations that oftentimes his name becomes shrouded, as it were, in mystery, and his teachings are credited with an authority that few dare question. Such were Plato and Aristotle among the Greeks; in the Middle Ages a legendary figure, Basil Valentine, as tradition has it a Benedictine monk, though his name cannot be found in any of the monastic records of the time, the author of a series of works written in the High German dialect of the end of the fifteenth and the beginning of the sixteenth century.

There is much that is obscure in the story of his life, and, like Homer, many towns claim the honour of his birth. If internal evidence is to be accepted, there can be no doubt that he spent his days in a monastery, and a story is told of him that throws an interesting light upon the man himself. Engaged upon experiments with antimony (his work, "The Triumphal Chariot of Antimony," had an influence upon medical practice down to the nineteenth century) he chanced one day to throw some out of his laboratory into the monastery yard. Here a herd of pigs gobbled it up with other refuse, much to Valentine's distress, who naturally feared the worst. But, observing the swine with care, he found that, after a preliminary period of digestive disturbance, they proceeded to eat voraciously and soon became fatter than any of the others. Having thus, so to speak, tested the physiologic action of the drug, it occurred to him that some of the brethren, who were noticeably frail and delicate, would be benefited by its administration. Accordingly he introduced surreptitiously some of the salts into their food. But, contrary to expectation, they suffered only from the more unpleasant results; some indeed, according to a less authentic version, actually perished. Valentine, therefore, named the substance antimony—that is, opposed to monks, since, though excellent for pigs, it was a form of monk's bane.

Amongst his other discoveries were the production of hydrochloric acid from salt and sulphuric acid, or oil of vitriol, and a method of obtaining copper from iron pyrites. In his numerous works there is to be found a mass of observations upon Nature, and more particularly on the action of drugs. In these days of tabloid remedies, his complaint that doctors did not know the composition or appearance of the medicines they prescribed has a curiously modern ring: "And whensoever I shall have occasion to contend in the school with such a Doctor, who knows not himself how to prepare his own medicines, but commits that business to another, I am sure I shall obtain the Palm from him. For, indeed, that good man knows not what medicines he prescribes to the sick; whether the colour of them be white, black, gray, or blue, he cannot tell; nor doth this wretched man know whether the medicine he gives be dry or hot, cold or humid; but he only knows that he found it so written in his books, and then pretends to knowledge, or as it were Possession by Prescription of a very long time. . . . Here again let it be lawful to exclaim, Good God, to what a state is the matter brought!"

The Travelling Scholars.

Few realise the travel that was undertaken in those days for purposes of study. Italy was then the home of post-graduate work, and all who wished for ampler opportunities for dissection willingly undertook the dangers and hardships of a journey across the Alps. As was told in last year's Harveian oration, all students intending to apply for professorial posts at Caius College, Cambridge, were almost bound to visit for a year or more the Universities of Padua and Bologna.

A beautiful story is recorded of Linacre, the founder of the Royal College of Physicians, who visited Italy more than a decade before the great Vesalius was born. After his course of study, which then included, as we have already seen, three years devoted to the arts, regretfully gaining the summit of the Alpine pass from which, far below, lay the last view of the sun-browned Italian plains, he paused, and, in true classic spirit, raised a little altar of stones in remembrance of his Alma Mater Studiorum. One quotation more, and we have done. Thus writes Nicholas of Cusa, Cardinal and Papal legate: "To know and to think, to see the truth with the eye of the mind is always a joy. . . As love is the life of the heart, so is the endeavour after knowledge and truth the life of the mind. In the midst of the movements of time, of the daily work of life, of its perplexities and contradictions, we should lift up our gaze fearlessly to the clear vault of heaven, and seek ever to obtain a firmer grasp of and a keener insight into the origin of all goodness and beauty, the capacities of our own hearts and minds, the intellectual fruits of mankind throughout the centuries, and the wondrous works of Nature around us." In such words as these do the Middle Ages find their ultimate expression.

